# Co-precipitation polymerization of dual functional monomers and polystyrene-*co*-divinylbenzene for ciprofloxacin imprinted polymer preparation†

**DOI:** 10.1039/d1ra05505d

**Published:** 2021-10-22

**Authors:** Huy Truong Nguyen, Nhat Thao Vuong Bui, Wilfried G. Kanhounnon, Kim Long Vu Huynh, Tran-Van-Anh Nguyen, Hien Minh Nguyen, Minh Huy Do, Michael Badawi, Ut Dong Thach

**Affiliations:** Faculty of Pharmacy, Ton Duc Thang University Ho Chi Minh City Vietnam nguyentruonghuy@tdtu.edu.vn h1600094@student.tdtu.edu.vn vuhuynhkimlong@tdtu.edu.vn nguyentranvananh@tdtu.edu.vn nguyenminhhien@tdtu.edu.vn thachutdong@tdtu.edu.vn +84 028 37 761 043; Laboratoire de Chimie Théorique et de Spectroscopie Moléculaire (LACTHESMO), Université d’Abomey-Calavi Benin gbedode.kanhounnon@fast.uac.bj; Faculty of Environmental and Food Engineering, Nguyen Tat Thanh University Ho Chi Minh City Vietnam dmhuy@ntt.edu.vn; Laboratoire de Physique et Chimie Théoriques UMR CNRS 7019, Université de Lorraine France michael.badawi@univ-lorraine.fr

## Abstract

Novel ciprofloxacin composite imprinted materials are synthesized by using co-precipitation polymerization of dual functional monomers (methacrylic acid and 2-vinylpyridine) and polystyrene-*co*-divinylbenzene. The intermolecular interactions between monomers and template are evaluated by molecular modeling analysis. The physicochemical properties of the obtained polymers are characterized using FT-IR, TGA, and SEM. Batch adsorption experiments are used to investigate adsorption properties (kinetic, pH, and isotherm). These polymers are employed to prepare the solid phase extraction cartridges, and their extraction performances are analyzed by the HPLC-UV method. DFT calculations indicate that hydrogen bonding and π−π stacking are the driving forces for the formation of selective rebinding sites. The obtained polymers exhibit excellent adsorption properties, including fast kinetics and high adsorption capacity (up to 10.28 mg g^−1^) with an imprinted factor of 2.55. The Scatchard analysis indicates the presence of specific high-affinity adsorption sites on the imprinted polymer. These absorbents are employed to extract CIP in river water with recoveries in the range of 65.97–119.26% and the relative standard deviation of 3.59–14.01%. Furthermore, the used cartridges could be reused at least eight times without decreasing their initial adsorption capacity.

## Abstract 2

Ciprofloxacin imprinted polymers were prepared using co-precipitation polymerization of methacrylic acid, 2-vinylpyridine and polystyrene-*co*-divinylbenzene.

## Graphical abstract

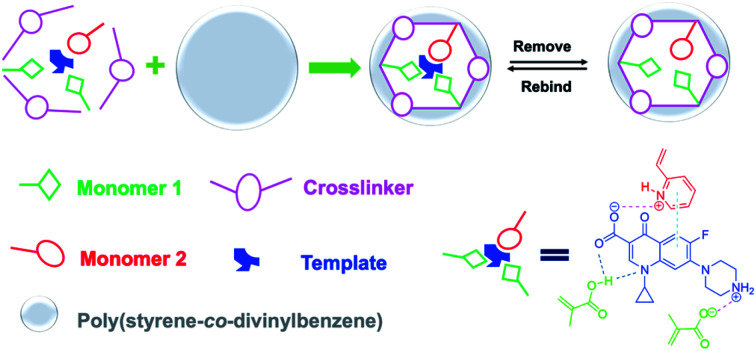

## Introduction

Molecularly imprinted polymers (MIP) are highly selective adsorbents synthesized *via* copolymerization of functional monomers and a crosslinker in the presence of target template molecules.^1^ These materials have attracted considerable research attention due to their valuable properties such as reproductivity, low cost, ease of preparation, and high selectivity toward target molecules.^2^ In fact, the imprinted materials have potential applications in numerous areas such as biomimetic sensors,^3–7^ biomimetic catalysts,^8^ drug delivery,^9^ protein crystallization,^10^ chromatography,^11^ and extraction.^12,13^

Ciprofloxacin (CIP), an important broad-spectrum fluoroquinolone antibiotic, has been approved for the treatment of certain infectious diseases in human and veterinary medicine. Yet, due to the misuse and abuse of CIP, there has been an increasing concern about the relevance of trace amounts of CIP in the environment. In the human body, only about 30% of intake CIP could be metabolized and the rest is excreted in original form *via* urine; CIP is thus expected to spread in the environment, in both wastewaters and soils.^14^ CIP imprinted materials have been developed to improve the extraction performance of trace CIP residues in biological and environmental matrices. Several molecularly imprinting technologies have been developed for synthesizing CIP imprinted materials, including bulk, precipitation, surface, and co-precipitation polymerization.

Bulk imprinting is a conventional technique for the preparation of CIP imprinted materials. The bulk polymerization is carried out from the mixture of all components (template, functional monomer, crosslinker, and initiator) in a low amount of porogen. After solvent extraction, grinding, and sieving steps, tiny irregular MIP particles (25–80 μm) are obtained. Even though simple, rapid, and pure MIP production can be produced without sophisticated instrumentation by the bulk polymerization, this technique also has its drawbacks: the loss of polymer particles, destruction of specific cavities, low adsorption capacity, low kinetic adsorption, and unsatisfactory resolution in chromatography.

To omit the post-polymerization steps and overcome their drawbacks, the CIP monolithic imprinted material was synthesized using graphene oxide and imidazolium ionic liquid monomer. The obtained MIP monolithic was employed to separate both CIP and levofloxacin from human urine with more than 93.8% recoveries.^15^ By changing precursor ratio and/or polymerization conditions of bulk imprinting, such as the porogen volume and solvent polarity, the spherical microsphere particles (0.1–0.3 μm) of imprinted polymer could be synthesized.^16–20^ The precipitation imprinted materials are potential candidates for selective stationary phase in HPLC.

Surface imprinting-based CIP-MIPS have been developed to increase mass transfer, binding capacity, and sorption kinetics. In this technique, a thin layer of imprinted polymer is covalently grafted on the surface of solid support. Functional yeast powder and magnetic graphene oxide embellished with mesoporous silica (MGO@mSiO_2_) were used as solid support for surface imprinting *via* atom transfer radical polymerization (ATRP) or free radical polymerization. These materials showed a high adsorption capacity (up to 27.02 mg g^−1^), fast kinetic adsorption, and good extraction performance. Their applications such as dispersive solid phase extraction (DSPE) or dispersive liquid–liquid microextraction (DLLME) have been investigated.^21,22^ The composite MIPs can be tailored for different purposes, including improving the physical and chemical stability of MIPs, functionalization of the proposed MIPs, increasing the specific surface area, and enhancing the adsorption capacity of the proposed MIPs.^23^ Zinc sulfide and polystyrene-*co*-divinylbenzene (PSD) were used to prepare CIP-MIPs *via* the co-precipitation technique. The obtained MIPs exhibit high adsorption capacity up to 19.96 mg g^−1^ with fast kinetic adsorption and good selectivity.^14^

In this study, we reported the synthesis of novel CIP composite MIPs by co-precipitating polymerization of dual functional monomers methacrylic acid (MAA) and 2-vinylpyridine (2-VP) under the presence of PSD. The combination of two functional monomer and PSD are expected to improve the selective extraction performances of the imprinted polymer. After characterizing the physiochemical properties (by FT-IR, TGA, and SEM) and sorption behaviours (kinetic, pH, and initial concentration), the synthesized polymers were further employed for its application in SPE. During the experiments, the solid phase extraction (SPE) procedures were optimized and subsequently applied for the extraction of CIP in river water samples collected from Tay Ninh province, Vietnam.

## Results and discussion

### Synthesis of imprinted polymers

The influences of reaction parameters on the adsorption properties of CIP imprinted polymers were summarized in Table 1. MIP1 and MIP2, synthesized from MAA functional monomer in MeOH : H_2_O (9 : 1, v/v) or ACN : H_2_O (8 : 2, v/v), have imprinted factors of 0.67 and 1.34 respectively. The highly polar aprotic solvent (ACN) is suitable for preparing of CIP imprinted polymers.^19,24^ In contrast, the polar protic solvent (methanol) reduces the imprinted polymer selectivity due to their competitive interaction with MAA *via* hydrogen bond.^19^ Moreover, the MIPs synthesized in highly polar solvents have small particles (<2 μm) for SPE applications.^17,25,26^

**Table 1:** Influence of reaction parameters on the adsorption capacity and selectivity of ciprofloxacin imprinted polymer

Polymer	Porogen (v/v)	CIP^a^ (mmol)	MAA^b^ (mmol)	2-VP^c^ (mmol)	EGDMA^d^ (mmol)	PSD^e^ (g)	*Q*_MIP_ f (mg g^−1^)	*Q*_NIP_ g (mg g^−1^)	IF^h^
MIP1	MeOH : H_2_O (9 : 1)	1.0	6.0	0.0	30.0	0.0	3.23	4.83	0.67
MIP2	ACN : H_2_O (8 : 2)	1.0	6.0	0.0	30.0	0.0	11.67	8.74	1.34
MIP3	ACN : H_2_O (8 : 2)	1.0	6.0	0.0	20.0	0.6	4.66	5.07	0.92
MIP4	ACN : H_2_O (8 : 2)	1.0	4.0	2.0	20.0	0.6	10.28	4.03	2.55

a CIP: ciprofloxacin.

b MAA: methacrylic acid.

c 2-VP: 2-vinylpyridine.

d EDGMA: ethylene glycol dimethacrylate.

e PSD: polystyrene-*co*-divinylbenzene.

f *Q*_MIP_: adsorption quantity of imprinted polymer.

g *Q*_NIP_: adsorption quantity of non-imprinted polymer.

h IF: imprinting factor = *Q*_MIP_/*Q*_NIP_.

Porogenic solvent completely dissolves all components (template molecule, functional monomer, crosslinking monomer, initiator) and creates macrostructures and morphology in the imprinted polymer.^17,27–29^ In this study, an aromatic nonpolar polymer (PSD) was employed as an ingredient for reducing solvent polarity and increasing the polymer particle size through the co-precipitation process. However, PSD could interfere with the intermolecular interaction between CIP and MAA. As a result, MIP3 displayed no adsorption selectivity toward the CIP molecule. Reportedly, the synergetic effect of multifunctional monomers increases significantly the selectivity.^30–32^ The complementary interactions between 2-VP and CIP *via* hydrogen bond, electrostatic, and π–π stacking are expected to improve greatly the adsorption selectivity.^33^ Therefore, 2-VP was used as a commentary functional monomer (with MAA) for synthesizing of MIP4. As shown in Table 1, MIP4 has a high CIP adsorption capacity up to 10.28 mg g^−1^ and an imprinted factor of 2.55. Thus, the combination of dual functional monomers and PSD led to high selectivity imprinted polymer with suitable particle size for SPE application. This imprinted polymer (MIP4), their corresponding non-imprinted polymer (NIP4) with high adsorption capacity and imprinting factor were used for further characterizations and application as selective adsorbent to extract of CIP in aqueous media.

### Monomer-template interactions

We first determine it by static DFT monomer–template interaction energies with many configurations. Interaction energies *E*_int_ were calculated with the below formula where *E*_cluster_, *E*_monomer_, *E*_template_ stand respectively for Energies of the system made of both monomer(s) and the template, of isolated monomer(s) and of the isolated template molecule.

1 *E*_int_ = *E*_cluster_ − (*E*_monomer_ + *E*_template_)

**Fig. 1:**
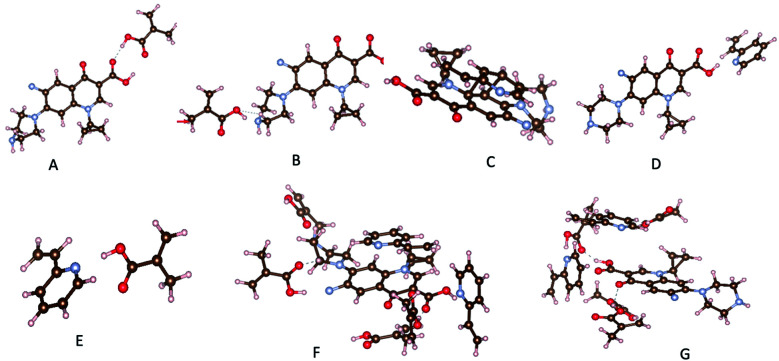
Most relevant interactions: (A) H⋯O for MAA-CIP, (B) H⋯N for MAA-CIP, (C) π–π stacking for 2-VP-CIP, (D) H⋯N for 2-VP-CIP, (E) H⋯N for MAA-2VP, (F) CIP-MIP, (G) CIP-NIP.

The results obtained for the interactions configurations between template and monomer screening are gathered in Tables S2 and S3.† They clearly indicated that, hydrogen bonds participate highly in the monomer–template interactions. Indeed, we found five favored configurations (Table S2†) where the MAA can bind to CIP molecule. Nevertheless, configurations with hydrogen bond between H atom and oxygen or nitrogen atoms are the most stable (Fig. 1A and B, Table S2,† configurations I, II and IV) as found by Gómez-Pineda and Quiroa-Montalván.^34^ For 2-VP, three stable configurations have been identified. The π–π stacking between aromatic ring of the template and monomers (Fig. 1C, Table S3,† configuration II) is the strongest interaction found, followed by hydrogen bonds. Hydrogen bonds occurred between hydrogen atom of hydroxyl group of the monomer and N atom of CIP molecule (Fig. 1D, Table S3,† configuration I) on one hand, and on the other hand between H atom linked to a nitrogen atom of the template molecule and the nitrogen atom of the monomer (Table S3† configuration V). The hydrogen atom of the vinyl group of the monomer interacts very weakly with the oxygen and nitrogen atoms of the template (Table S3,† configuration III and IV). It should be noticed that MAA interacts more strongly with the template comparatively to 2-VP. In the absence of CIP, monomers interact with each other. The hydrogen of the carboxylic group of MAA forms hydrogen bond with the nitrogen atom of 2-VP (Fig. 1E, Table S4,† configuration I). This fact may be deleterious for CIP adsorption given the fact that the number of interaction sites on monomers will decrease comparatively to that in MIP.

### Monomers arrangement in MIP and NIP

In a second step, a mixture of four MAA, two 2-VP and one template molecule was considered to mimic the experimental conditions. From the corresponding optimized structure, we deleted the template and optimized the remaining, the obtained structure holding for the imprinted copolymer (MIP). We also optimized system built of four MAA and two 2-VP without the template (NIP). The obtained structures are presented in Table S5.† It clearly appears in NIP structure (Fig. 1G, Table S5,† configuration II) that monomers sites which are supposed to react with the template, interact with each other, a fact that the occurring is limited in the MIP by the presence of the CIP molecule. In addition, the presence of template during copolymerization may create sharp suiting for CIP adsorption on polymer contrarily to the NIP (Table S5,† configurations I and II). Indeed, once monomers are bonded to the template in the most efficient way, the crosslinker binding to monomers will help keeping the sharp even after removal of the template. We then adsorb CIP on both MIP and NIP structures (Fig. 1F and G, Table S5,† configurations III and IV). Adsorption energy of CIP on both MIP and NIP polymers were calculated as follows:

2 *E*_ads_ = *E*_cluster_ − (*E*_MIP/NIP_ + *E*_template_)

where *E*_ads_ is the adsorption energy of CIP, *E*_cluster_ the energy of MIP or NIP and CIP system, *E*_template_ energy of CIP molecule. Negative adsorption energy means that interaction or adsorption is favored. Adsorption energies on both polymers are negative indicating that CIP may adsorb on NIP and MIP. CIP adsorption energy on MIP (−191 kJ mol^−1^) in our calculations conditions is at least twice higher in absolute value than that on NIP (−72 kJ mol^−1^) showing the higher selectivity of MIP toward CIP comparatively to NIP. Even though, we reported here out-solvent template–monomer interactions, convenient solvent like the one identified in the experimental work will corroborate or even enhance the theoretical findings. Finally, knowing that CIP molecule can easily form hydrogen bonds, protic solvents should not be used to avoid competition with monomers adsorption.

### Characterization

#### FT-IR study

**Fig. 2:**
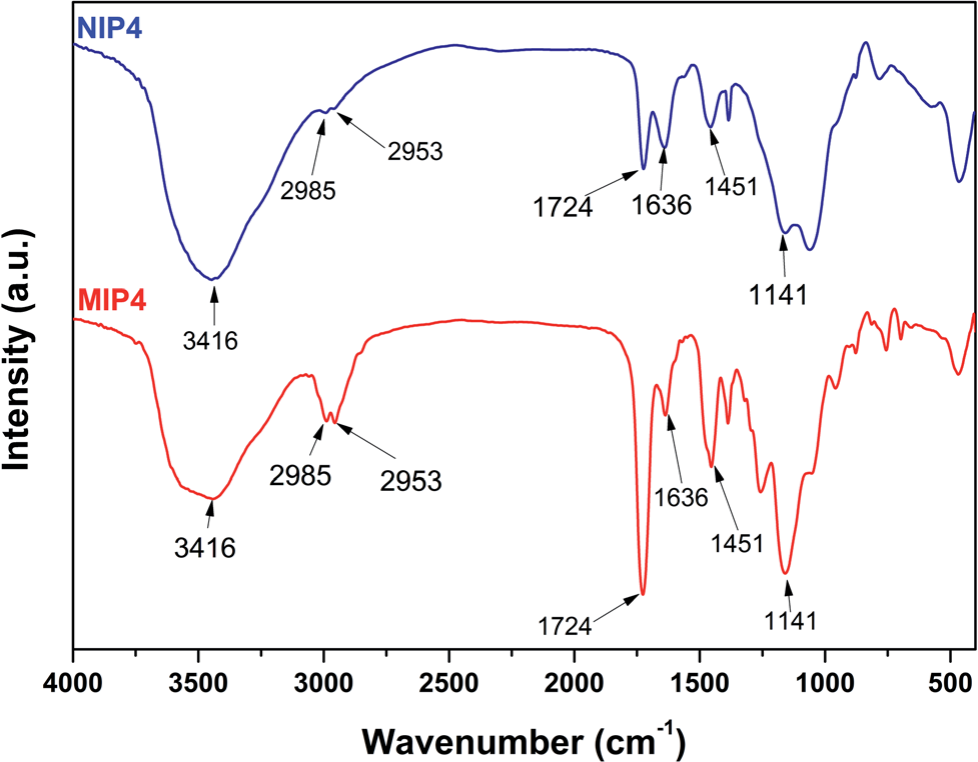
FT-IR spectra of MIP4 and NIP4.

The chemical structure of the polymers was characterized by FT-IR spectra. As shown in Fig. 2, MIP4 and NIP4 displayed identical absorption peaks. The broad and strong absorption peak around 3416 cm^−1^ was characteristic of the vibration of O–H bond. The weak absorption peaks that appeared at 2985 and 2953 cm^−1^ were due to the symmetric and asymmetric stretching vibration of the saturated C–H bond. The narrow and strong signal of C=O vibration was observed at 1724 cm^−1^.^35^ The characteristic absorption peaks of 2-VP appeared at 1637 and 1450 cm^−1^ were due to the C=N and C=C stretching vibration of the pyridine ring.^32^ The strong absorption peak at 1141 cm^−1^ was characteristic of the stretching vibration of C–O bond. These results confirmed the presence of MAA, 2-VP, and EDGMA in the synthesized polymers through a successful copolymerization process. Furthermore, the FT-IR of unleached and leached imprinted polymer show similar pattern, which is referred to the similar backbone of the polymer samples (see ESI, Fig. S1†). The results indicated that the imprinted polymer was remained stable after template elimination process using acid solution.^13,36,37^

#### Thermogravimetric analysis

**Fig. 3:**
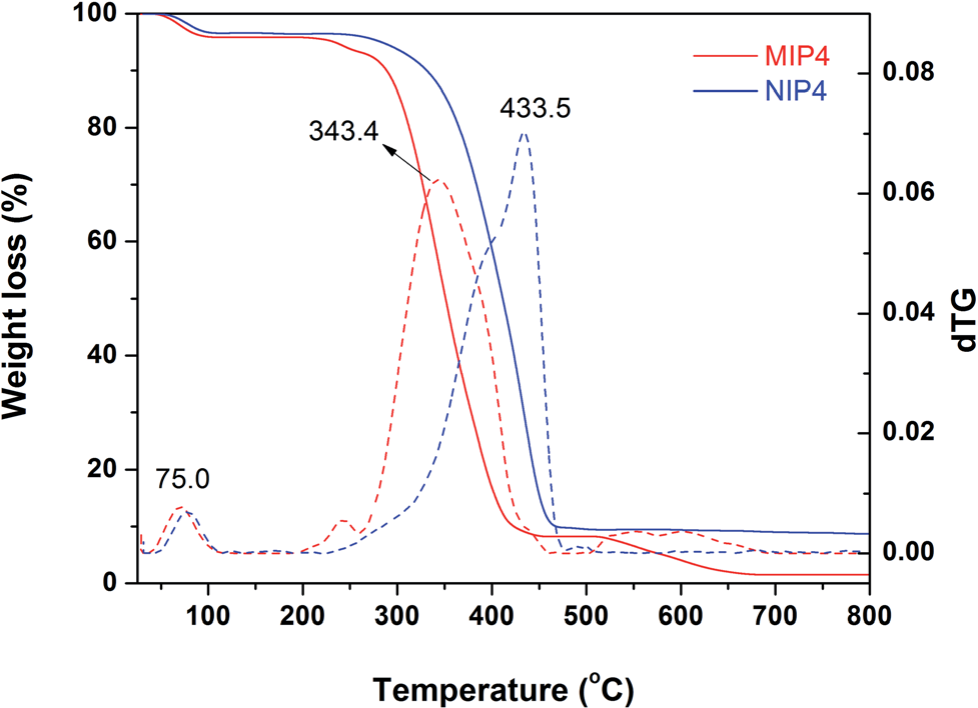
TGA analysis (solid) and dTG plot (dash) of MIP4 and NIP4.

The TGA curve and dTG plot of MIP4 and NIP4 are shown in Fig. 3. It could be seen that the imprinted polymer and their corresponding non-imprinted polymer exhibited different thermal profiles. For MIP4, the weight loss (4%) below 100 °C was due to the loss of entrapped solvent and water. The principal weight loss (85.5%) occurred from 200 to 460 °C, corresponding to the decomposition of the polymer backbone. The polymer was totally decomposed at 660 °C. The weight loss (3.4%) at a temperature below 100 °C observed for NIP4 was due to adsorbed water and residue solvent evaporation. The degradation of the polymer backbone started at 200 °C and totally decomposed at 470 °C. The dTG plot showed an identical weight loss occurred at around 75 °C for both MIP4 and NIP4 *via* the evaporation of the volatile organic solvent (ACN, MeOH). However, the principal decomposition occurred at 343.4 °C for MIP4, while NIP4 has a maximum decomposition temperature at 433.5 °C. These results suggested that MIP4 and NIP4 have differences in chemical composition and/or macromolecular structure. During the synthesis process, it was observed that the precipitation rate in MIP4 synthesized mixture is about two times faster than in NIP4. The rapid polymerization kinetic under the presence of CIP could lead to the formation of low molecular weight polymer during the imprinted process. These features could explain that MIP4 has a lower maximum decomposition temperature and a higher complete decomposition temperature than NIP4.^38^

#### Surface morphology

**Fig. 4:**
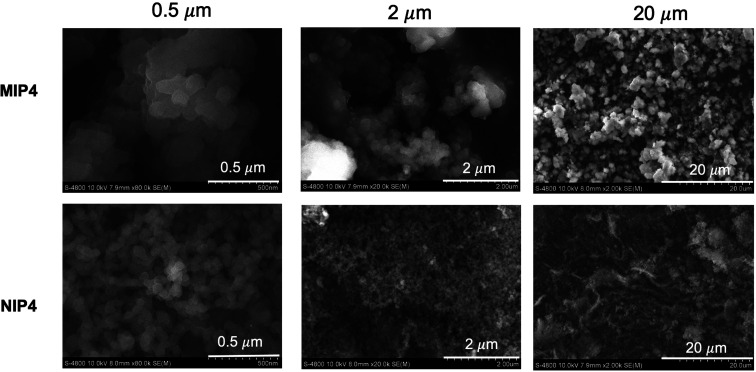
SEM images of MIP4 and NIP4.

The difference in surface morphology of MIP4 and NIP4 was observed by scanning electron microscopy (Fig. 4). In general, MIP4 has a larger particle size than NIP4. MIP4 has a rough, porous surface morphology with the aggregate structure of irregular particles. In comparison, NIP4 has a rough, porous surface morphology with the aggregate structure of spheric polymer particles. The effect of CIP on surface morphology of MIP has been reported previously.^16,17,19,39,40^

### Adsorption study

#### Kinetic adsorption

**Fig. 5:**
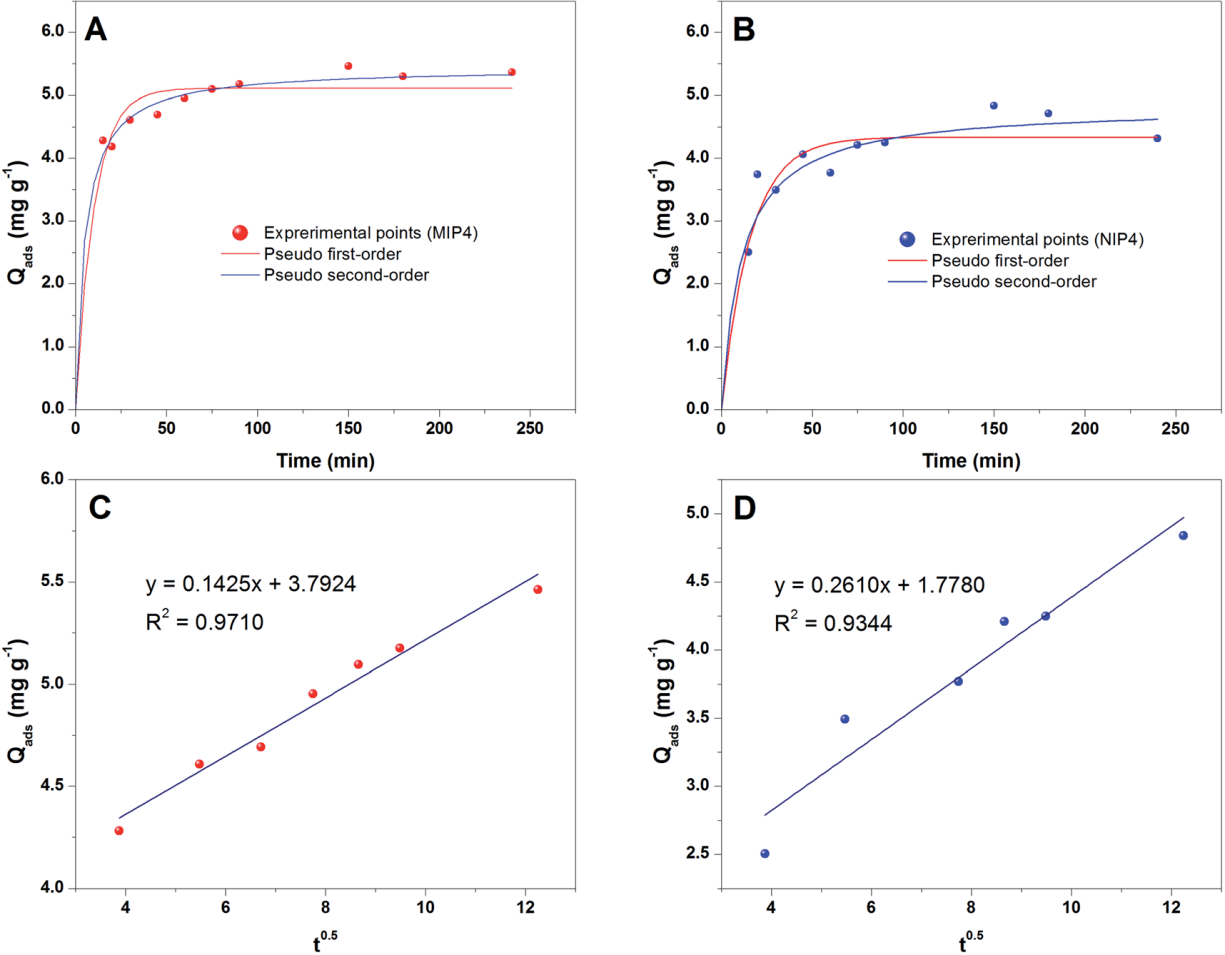
Adsorption kinetic analysis: the fit from the pseudo first-order and second-order model for MIP4 (A) and NIP4 (B); the fit from the Weber's interparticle diffusion model for MIP4 (C) and NIP4 (D). Kinetic adsorption condition: 10 mg of polymer, pH: 6.7, adsorption solution: 20 mL of 5 mg L^−1^ CIP in deionized water, temperature 30 ± 0.5 °C, shaking time: 15–240 min.

The effect of contact time on CIP sorption onto MIP4 and NIP4 was shown in Fig. 5. The binding rate of MIP4 is very fast in the first 15 min. It reaches 90% of its maximum value after 25 min and then increases slowly with the shaking time. Similar results were observed for NIP4. Completed sorption equilibria were reached after 150 min. These results indicated rapid kinetic adsorption than other rebinding processes, *e.g.*, CIP adsorption onto bulk imprinted polymers.^22,25,41^

To investigate the binding mechanism in imprinted polymers, different kinetic models were applied. The Lagergren pseudo-first-order, the pseudo-second-order and the Weber's intraparticle diffusion model can be expressed as eqn (3)–(5), respectively:

3 *q*_*t*_ = *q*_e (1 − e_^*k*_1_*t*^_)_,

4
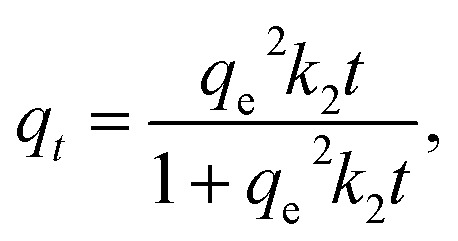

5 *q*_*t*_ = *k*_id_*t*^0.5^ + *C*,

where, *q*_*t*_ and *q*_e_ are the amount of adsorbed CIP at equilibrium and at time *t*; *k*_1_ is the equilibrium rate constant in pseudo-first-order; *k*_2_ is the equilibrium rate constant in pseudo-second-order and *k*_id_ is the equilibrium rate constant in the Weber's intraparticle diffusion model.

The fitted results according to the pseudo-first-order and pseudo-second-order models for MIP4 and NIP4 are shown in Fig. 5A and B, respectively. The kinetic adsorption parameters and nonlinear regression correlation coefficient (*R*^2^) values were calculated (see ESI, Table S1†). A high value of *R*^2^ (0.9312–0.9740) for the pseudo-first-order kinetics suggested that this model can be used to represent the kinetic adsorption of CIP onto the polymers. However, the *R*^2^ values (0.9391–0.9926) for pseudo-second-order model are slightly higher than those for the pseudo-first-order. The pseudo-second-order model is the best fit model for the experimental data of adsorption kinetic. These results indicated that the chemical process was the rate-limiting step in this adsorption kinetic process.^22^

The rate constant *k*_2_ obtained for the second-order model is 0.0361 and 0.0183 mg g^−1^ min^−1^ for MIP4 and NIP4, respectively. The rate of MIP4 has a higher rate constant *k*_2_ than that of NIP4 due to the higher adsorption affinity, capacity, and selectivity of MIP4 toward CIP molecule. A similar tendency was also observed for the pseudo first-order model. The above results indicated the presence of specific binding sites on the imprinted polymer MIP4.

The plots of *q*_*t*_*versus t*^0.5^ should represent straight lines and were used to obtain the rate constants. As shown in Fig. 5C and D, the kinetic plots following Weber's intraparticle diffusion model do not pass through the origin. The results indicates that the intraparticle diffusion is the rate controlling step.^42,43^

#### Influence of pH

CIP exists in three ionic forms in aqueous solution (see ESI, Fig. S2†). Cationic form CIP^+^ is the dominant species at pH below 5.90. From pH 5.90 to 8.89, CIP can exist as zwitterionic (CIP^±^) and neutral forms (CIP°). The zwitterionic form CIP^±^ is the dominant species resulting from the charge balance of the deprotonated carboxylic acid group and protonated from the secondary amine on the piperazine group.^44,45^ Whereas CIP occurs in its anionic carboxylate form at pH >8.90. The effect of pH on the adsorption behaviour is usually due to the formation of ionic species of adsorbate and the surface charge of the adsorbent.

**Fig. 6:**
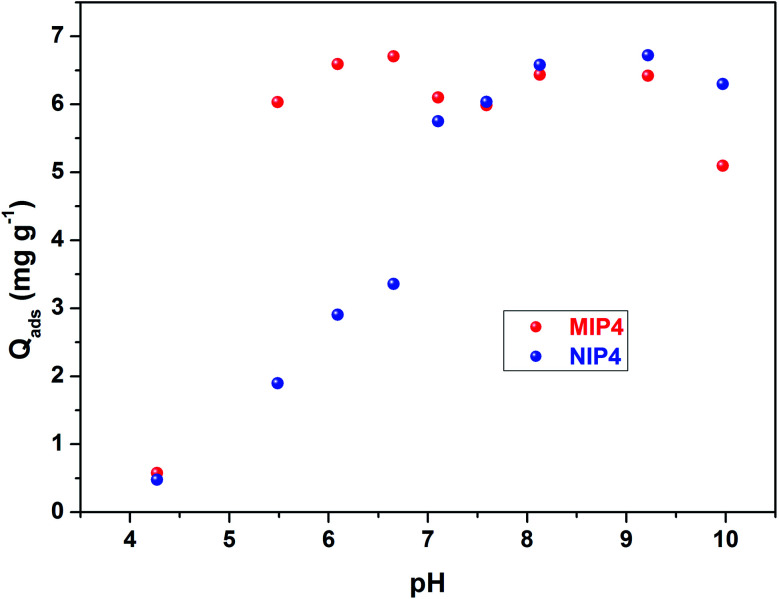
Effect of pH on the adsorption properties of MIP4 and NIP4. Adsorption condition: 10 mg of polymer, pH: 4–10, adsorption solution: 20 mL of 5 mg L^−1^ CIP in deionized water, temperature: 30 ± 0.5 °C, shaking time: 3 h.

For imprinted polymers, solution pH affects both the adsorption capacity and the selectivity. Fig. 6 shows the effect of pH on the adsorption capacity of CIP on MIP4 and NIP4. It could be seen that MIP4 and NIP4 have differences in CIP adsorption properties due to pH. The adsorption process is low at pH below 5. At pH >5, the adsorption capacity of MIP4 significantly increases at elevated pH and reaches the maximum value at pH ∼6.5. In contrast, the adsorption capacity of NIP4 increases slower than that of MIP4 and reaches the maximum value at pH ∼9. The adsorption properties of MIP4 and NIP4 are identical for between pH 7–9. When pH >10, the sorption capacity decreases with the increase of pH. This could be due to the competition adsorption of CIP^−^ and OH^−^.^43^ Moreover, the weak sorption at extremely low and high pH ranges can be suggested by the great electrostatic repulsion between the ionic species of CIP and the adsorbent surface.^44^ It is important to note that MIP4 exhibits adsorption selectivity for CIP only in the pH range between 5–7. This could be due to the imprinting process being carried out in the weak acid medium of MAA. Thus, the rebinding experiments must proceed in this pH range to obtain the best selective extraction performance.

#### Adsorption isotherm

**Fig. 7:**
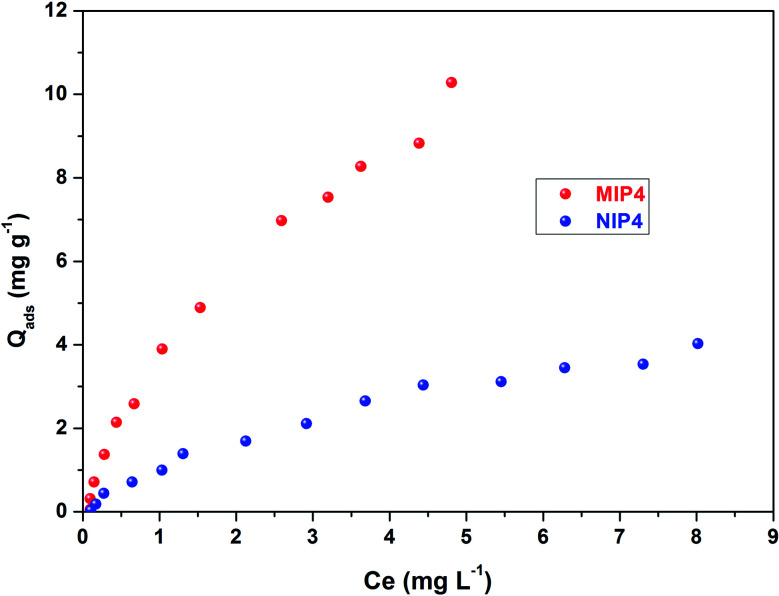
Adsorption isotherms of MIP4 and NIP4. Adsorption condition: 10 mg of polymer, pH: 6.7, adsorption solution: 0.12–10.10 mg L^−1^ CIP in deionized water, temperature 30 ± 0.5 °C, shaking time: 3 h.

The rebinding amount is an essential property of the imprinted materials. The adsorption isotherms of CIP onto the obtained polymer were studied using adsorption experiments obtained from the previously determined optimized adsorption condition (shaking time ≥3 h, neutral initial pH 6.7, without HCl or NaOH). The adsorption isotherms of CIP onto MIP4 and NIP4 are shown in Fig. 7. The amount adsorbed on MIP4 significantly enhanced with the increase of initial CIP concentration, while that of NIP4 has a slight increase. This indicated the amount of imprinted polymer has a higher adsorption affinity to CIP than that of non-imprinted polymer. Furthermore, the adsorption capacity of MIP4 was much higher than that of NIP4 over the whole tested concentration range. When the equilibrium concentration of adsorption solution reaches, the maximum adsorption capacity of MIP4 (10.28 mg g^−1^) was 2.55 times higher than NIP4 (4.03 mg g^−1^).

The maximum binding capacity and the dissociation constant were employed to evaluate the binding properties of the MIP. These properties are usually calculated using the Scatchard model,^29,39,46,47^ which can be expressed as eqn (6):6
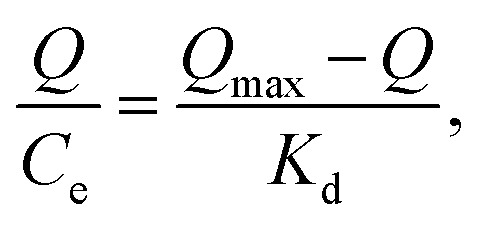
where, *Q* is the amount adsorbed of CIP onto MIP, *Q*_max_ is the maximum adsorption capacity, *C*_e_ is the CIP concentration at equilibrium, and *K*_D_ is the dissociation constant.

**Fig. 8:**
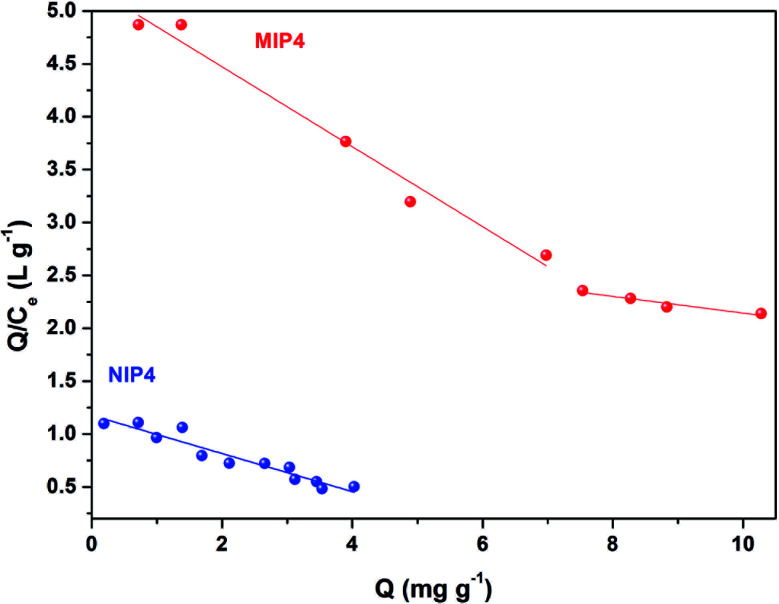
Scatchard analysis for MIP4 and NIP4.

As shown in Fig. 8, the Scatchard plot of MIP4 shows an apparent nonlinear relationship, while the linear relationship was obtained for the whole graph of NIP4. For MIP4, the two distinct sections at both ends of the graph that are good linear relationships, suggesting that the binding sites in the imprinted polymer are heterogeneous regarding the affinity of CIP.^25,29^ It would be reasonable to assume that the binding sites can be classified into two distinct groups with different specific properties. There was various interaction between functional monomers with CIP molecules, and the interaction forms many kinds of complexes that have binding sites with different components.^25^ The data can be fitted according to the sections of the linear relationship. The respective *Q*_max_, *K*_D_, and linear regression correlation coefficients (*R*^2^) were shown in Table 2.

**Table 2:** The results of Scatchard analysis

Polymer	Binding sites	*Q*_max_ (mg g^−1^)	*K*_D_	*R* ^2^
MIP4	Higher affinity	13.8346	2.6462	0.9800
	Lower affinity	37.1723	12.6743	0.9292
NIP4	—	6.5308	5.5525	0.9207

### Solid phase extraction study

#### Optimization of the extraction conditions

Variables influencing extraction efficiency, including amount of MIP sorbent and type of eluent solvent, were investigated.

The amount of MIP sorbent greatly influences the recovery. In this research, the effect of different amounts of MIP (5, 10, 20, and 30 mg) was evaluated. As shown in ESI Fig. S3,† the extraction recovery increases slowly with the increase of the MIP amount from 5 to 20 mg and remains stable when the MIP amount reaches 30 mg. Therefore, 20 mg of MIP was prepared for SPE cartridges and utilized in the following experiments.

The type of eluent is the key factor to the extraction efficiency, several types of eluent including MeOH : AcOH (9 : 1, v/v), MeOH : AcOH : H_2_O (85 : 15 : 5, v/v/v), ACN : AcOH 0.03% (2 : 8, v/v) and ACN : HCOOH 0.03% (2 : 8, v/v) were investigated to obtain the optimized condition. The result showed the extraction efficiency of MeOH : AcOH : H_2_O (85 : 15 : 5, v/v/v) and ACN : HCOOH 0.03% (2 : 8, v/v) were higher than that of MeOH : AcOH (9 : 1, v/v) and ACN : AcOH 0.03% (2 : 8, v/v) (see ESI Fig. S3†). For convenience, ACN : HCOOH 0.03% (2 : 8, v/v) was selected as the optimal eluting solvent for the subsequent studies.

#### Reusability performance of MIP

**Fig. 9:**
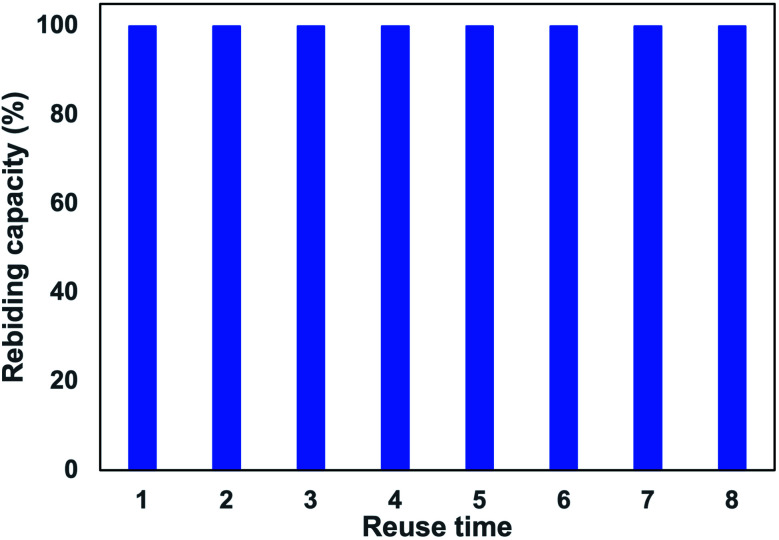
Recycle performance of MIP-SPE cartridge for CIP extraction. Recycle condition: 20 mg of polymer, loading: 3 mL of CIP 0.1 μg L^−1^, washing: 3 mL of deionized water, eluting: 3 mL of ACN : HCOOH 0.03% (2 : 8, v/v), recycling: 15 mL MeOH : AcOH (9 : 1, v/v).

Reusability is an important parameter used to test the performance of applied MIP sorbent. The after-used cartridges were regenerated and reused to adsorb CIP in a subsequent cycle. The results of the reusability experiment on MIP for CIP with eight adsorption and desorption iterations were shown in Fig. 9. It showed that MIP4 could be effectively regenerated for further use without a significant loss (less than 1%) of initial binding capacity after eight cycles. This indicated that MIP sorbent is retaining good CIP sorption capacity after multiple uses. The reusability performance of the as-presented sorbent is comparable or even better compared to that of bulk^40^ or surface imprinted polymerization techniques.^14,22^

#### Application in the real sample

To evaluate the application of the developed MIP-SPE method, it was applied to analyze the target analytes in real sample. It was found that none of the target analyte was detected in the real water samples. Furthermore, the unidentified peaks (at retention time from 2 to 4 min) on initial river sample were not observed on all chromatograms of SPE extracted solution. Therefore, it is reasonable to assume that the MIP-SPE cartridges could efficiently purify and separate CIP from the impurities of river water collected (see ESI, Fig. S4†).

To determine the accuracy, the obtained samples were spiked with the different concentrations of CIP (from 0.0005 to 0.11 mg L^−1^). The recoveries for the samples are illustrated in Table 3. The CIP concentration of eluate obtained from spiked samples from 0.0005 to 0.005 mg L^−1^ cannot be determined due to these concentrations being under the limit of quantification of the developed method. Therefore, a further enrichment process (and other analytical methods of higher sensitivity) is necessary to determine CIP at low concentrations. The average recoveries for the CIP at 0.01 and 0.1 mg L^−1^ were in the range of 65.97–119.26% with the relative standard deviation (RSD) values of 3.59–14.01%. It was found that the CIP extraction recovery decreased with increasing CIP concentration. This could be due to the competitive adsorption of matrix samples or lack of eluting volume. Thus, further research will be carried out to apply the abovementioned imprinted polymer in large scale and for various sample types.

**Table 3:** CIP extraction recovery from river samples at different concentration (SPE condition: 20 mg of polymer, loading: 3 mL of sample, washing: 3 mL of deionized water, eluting: 3 mL of ACN : HCOOH 0.03% (2 : 8, v/v), *n* = 3)

Sample	Added (mg L^−1^)	Founded (mg L^−1^)	Recovery (%)	RSD (%)
River water	0.000	ND^a^	ND	ND
	0.0005	<0.010	—	—
	0.001	<0.010	—	—
	0.011	0.0131	119.10	14.01
	0.050	0.0375	75.10	3.59
	0.106	0.0699	65.97	4.55

a ND: not detected.

## Experimental

### Chemicals

Ciprofloxacin hydrochloride (CIP.HCl, 95%) was obtained from Zhejiang Guoabang Pharmaceutical Co., Ltd, China. Methacrylic acid (MAA, 99%), 2-vinylpyridine (2-VP, 97%), ethylene glycol dimethacrylate (EDGMA, 98%), and azobisisobutyrontrile (AIBN) 12% wt in acetone, and poly(styrene-*co*-divinylbenzene), 200–400 mesh particle size, 2% cross-linked (PSD) were purchased from Sigma-Aldrich, USA. Acetonitrile, methanol, acetic acid, formic acid, and triethylamine with HPLC grade were purchased from Merck, USA. The standard stock of CIP (500 mg L^−1^) was prepared in deionized water, and the working solutions were diluted from the stock solution with deionized water. The standard solutions were stored at 4 °C to be stable for one month. All chemical reagents were used as received without further treatment.

### Methods

#### Preparation of imprinted polymer

The imprinted polymers were synthesized by the co-precipitation polymerization technique.^14^ Briefly, CIP (0.169 g, 0.5 mmol), methacrylic acid (0.173 mL, 2 mmol), 2-vinylpyridine (0.11 mL, 1 mmol), and 50 mL of porogen ACN : H_2_O (8 : 1, v/v) were added into a brown screw-capped glass bottle. The mixture was sonicated for 15 min to get the homogenous solution. Then, PSD (0.3 g) was added to the above solution and shacked at 30 °C at 200 rpm for 4 h using a Stuart Shaking Incubator. Next, ethylene glycol dimethacrylate (1.925 mL, 10 mmol) and azobisisobutyronitrile (20 mg) were added to the mixture. The oxygen in the bottle was removed by argon for 15 min. The polymerization was initiated at 60 °C for 24 h in the thermostatic water bath. The CIP template was eliminated by repeated washing with MeOH : AcOH (9 : 1, v/v) in an ultrasonic bath, and HPLC-UV was then used to detect CIP. Finally, the obtained polymer particles were dried at 110 °C for 6 h. The corresponding non-imprinted polymer (NIP) preparation without the CIP template was like this protocol.

#### Theoretical study

DFT calculations have been performed using the Quickstep module^48^ of the CP2K simulation package^49^ to investigate the interactions between ciprofloxacin and two monomers, methacrylic acid and 2-vinylpyridine. The use of hybrid Gaussian and plane wave (GPW) method^50^ and Geodecker–Teter–Hurter (GTH) relativistic pseudo potentials that provide a compact and efficient description of core electrons^51^ make this methodology very efficient and accurate to describe molecular interactions.^52–54^ The PBE functional^55^ with Grimme dispersive correction method D3 (ref. 56) was used along with the DZV2P-MOLOPT basis set^57^ for all atoms. The plane-wave cut-off energy has been set to 300 Ry to achieve ionic force convergence.^53^ The *ab initio* molecular dynamics simulations were performed in the canonical (NVT) ensemble with a time step of 0.5 fs. The temperature was set to 300 K by the Generalized Langevin Equations (GLE) thermostat. Three-dimensional periodic boundary conditions were applied. A large supercell of 22 × 22 × 22 Å was used to avoid artefact interactions between periodic images of cell and allows us to sample the Brillouin zone with the gamma-point. To catch the best possible interaction configurations, we use both static DFT relaxations based on various guess structures and 10 ps of *ab initio* molecular dynamics allowing to explore more deeply the configurational space.^58,59^

#### Polymer characterization

The FT-IR spectroscopy analyses were conducted by ATR-FTIR FT/IR6600A spectrometer, Seri A012761790. Thermogravimetric analysis (TGA) was carried out using a TG-DSC LabSys Evo 1600, SETARAM. The samples were heated from 25 to 800 °C at a heat rate of 10 °C min^−1^ under nitrogen atmosphere. Scanning electron microscopy (SEM) was recorded using SEM S-4800, 10 kV, 7.9 mm.

#### High performance liquid chromatography method

The separation was conducted on HPLC Agilent 1260 Infinity II system using a Phenomenex Luna C18 column (150 mm × 4.6 mm. i.d., 5 μm) eluted by gradient mobile phase program consisting of 0.1% formic acid (v/v) in water (A) and 0.1% formic acid (v/v) in acetonitrile (B) as follows: 0–10 min, 10–20% B; 10–15 min, 20–90% B; 15–20 min, 90% B; 20–25, 90–10% B; 25–30, 10% B. The flow rate was set at 0.8 mL min^−1^, and the injection volume was 20 μL. The column temperature was maintained at 30 °C. And the UV detector was monitored at 270 nm.

#### Quantification method

Ciprofloxacin was identified by comparing its retention times to that of ciprofloxacin in standard solution. To calculate the calibration curve of ciprofloxacin, standard stock solution was prepared and then subsequently diluted to different concentrations. Each concentration was analyzed in triplicate. The calibration curve was constructed by plotting the peak area with the corresponding standard concentration, while the linearity of the calibration curve was evaluated by correlation coefficients (*R*^2^). The limit of detection (LOD) and limit of quantification (LOQ) of the quantification method were determined based on signal to noise ratio (S/N), in which for LOD, a S/R of 6 : 1 is usually used and, while for LOQ, a S/N of 10 : 1 is used (see ESI, Fig. S5 and S6†). As a result, the characteristic parameters of quantification method are as follows: calibration curve: *y* = 113.2500*x* + 1.2810, *R*^2^ = 0.9994, linearity range: 0.005–0.500 mg L^−1^, LOD = 0.001 mg L^−1^, LOQ = 0.01 mg L^−1^.

#### Adsorption study

The individual adsorption isotherms of CIP onto the polymer were determined by batch experiments. The kinetic studies were carried out by equilibrating 10 mg of polymer and 20 mL of 5 mg L^−1^ CIP solution at neutral pH 6.7 (without modification) for different intervals from 15 to 240 min. The effect of pH was studied by shaking 10 mg of polymer with 20 mL of 5 mg L^−1^ CIP solution at pH 4–10 for 3 h; To adjust the pH value, 0.1 M HCl and 0.1 M NaOH were used. The adsorption isotherms were determined by shaking 10 mg of polymer in 20 mL of CIP solution at neutral pH for 3 h. The initial CIP concentrations were varied from 0.12 to 10.0 mg L^−1^. All adsorption tests were shaken at 30 ± 0.5 °C in a Stuart Shaking Incubator. The supernatants were filtered out by 0.45 μm nylon Millipore filters. The concentration of CIP in the supernatants was determined by HPLC-UV. The equilibrium adsorption capacity was calculated as follows:7
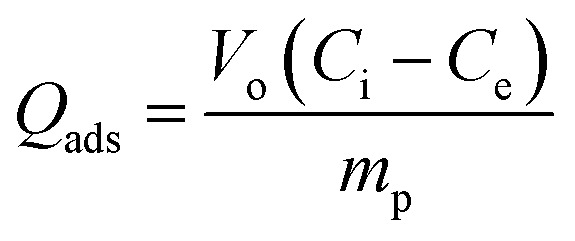
where *Q*_ads_ (mg g^−1^) is the equilibrium adsorbed amount of CIP, *C*_i_ and *C*_e_ (mg L^−1^) are the initial and final concentrations of the CIP solution, respectively; *V*_o_ (L) is the volume of the CIP solution; and *m*_p_ (g) is the mass of polymer.

#### Solid phase extraction study

To evaluate the applicability of the synthesized polymer, 20 mg of each MIP or NIP were packed between two frits into 3.0 mL empty solid phase extraction (SPE) cartridges. The cartridge was conditioned and equilibrated with 1 mL of MeOH and 1 mL of water. After that, 3 mL of CIP solution in water (0.1 mg L^−1^) was loaded onto the SPE cartridge, which was then washed with 3 mL of deionized water. Afterwards, the retained analytes were eluted with 3 mL of ACN : HCOOH 0.03% (2 : 8, v/v). All eluates were collected and properly evaporated or diluted and reconstituted before their HPLC-UV analysis.

#### Reusability study

The after-used SPE cartridges were regenerated by washing with 15 mL MeOH : AcOH (9 : 1, v/v) and reused for novel SPE procedures.

#### Sample preparation

Water samples were collected from the Tay Canal (11°21′53.2′′N 106°07′21.5′′E), Tay Ninh province, Vietnam. The samples were collected in pre-cleaned amber glass bottles and immediately transported to the laboratory for the analysis. The obtained samples were filtered through 0.45 μm filters and loaded onto MIP-SPE cartridges and analyzed as described above. These samples were spiked with different levels of CIP because they did not show detectable signals of CIP.

#### Statistical analysis

The descriptive statistic was applied to describe the basic features of the data in this research. All data presented in this study are expressed as the mean of at least three independent experiments.

## Conclusions

The co-precipitation imprinting technique was used for synthesizing composite CIP imprinted materials. MIP with high adsorption capacity and selectivity was obtained through co-precipitation polymerization of dual functional monomers (MAA and 2-VP) and PSD. The multi-intermolecular interactions (hydrogen bond and π−π stacking) are responsible for the formation of selective adsorption sites. The physicochemical characterization indicated that the CIP template affected the thermal properties and surface morphology of the imprinted materials. The composite imprinted polymer has excellent adsorption properties, including fast kinetic adsorption, high adsorption capacity, and selectivity, together with the presence of specific rebinding sites. Furthermore, the MIP-SPE cartridges could be regenerated and reused at least eight times without significantly decreasing their adsorption capacity. It could be employed to extract CIP from collected river samples with the recoveries from 65.97–119.26% with the RSD values of 3.59–14.01%, hence implying its potential use in detecting trace amount of CIP.

## Author contributions

Conceptualization, U. D. T., W. G. K.; methodology, U. D. T., W. G. K.; validation, H. T. N., K. L. V. H. M. B., V. A. N. T.; investigation, N. T. V. B., W. G. K.; writing—original draft preparation, U. D. T., W. G. K., M. H. D., H. T. N.; writing—review and editing, H. M. N, M. B.; project administration, U. D. T.; funding acquisition, U. D. T. All authors have read and agreed to the published version of the manuscript.

## Conflicts of interest

There are no conflicts to declare.

## Supplementary Material

RA-011-D1RA05505D-s001
