# Supplementary material for: Co-precipitation polymerization of dual functional monomers and polystyrene-co-divinylbenzene for ciprofloxacin imprinted polymer preparation
Source: RSC Adv. 2021 Oct 22;11(54):34281–90. doi: 10.1039/d1ra05505d (PMC9042346; doi:10.1039/d1ra05505d)
Supplement: RA-011-D1RA05505D-s001 [file RA-011-D1RA05505D-s001.pdf]

Electronic Supplementary Material (ESI) for RSC Advances.
This journal is © The Royal Society of Chemistry 2021

Electronic Supporting Information for

Co-precipitation polymerization of dual functional monomers and polystyrene-*co*-divinylbenzene for ciprofloxacin imprinted polymer preparation

Huy Truong Nguyen,[a] Nhat Thao Vuong Bui,[a] Wilfried G. Kanhounnon,[b] Kim Long Vu Huynh,[a] Tran-Van-Anh Nguyen,[a] Hien Minh Nguyen,[a] Minh Huy Do,[c] Michael Badawi,[d] and Ut Dong Thach*[a]

[a.] Faculty of Pharmacy, Ton Duc Thang University, Ho Chi Minh City, Vietnam

[b.] Laboratoire de Chimie Théorique et Spectroscopie Moléculaire (LACTHESMO), Université d'Abomey-Calavi, Bénin

[c.] Faculty of Environmental and Food Engineering, Nguyen Tat Thanh University, Ho Chi Minh City, Vietnam

[d.] Laboratoire de Physique et Chimie Théoriques UMR CNRS 7019, Université de Lorraine, France

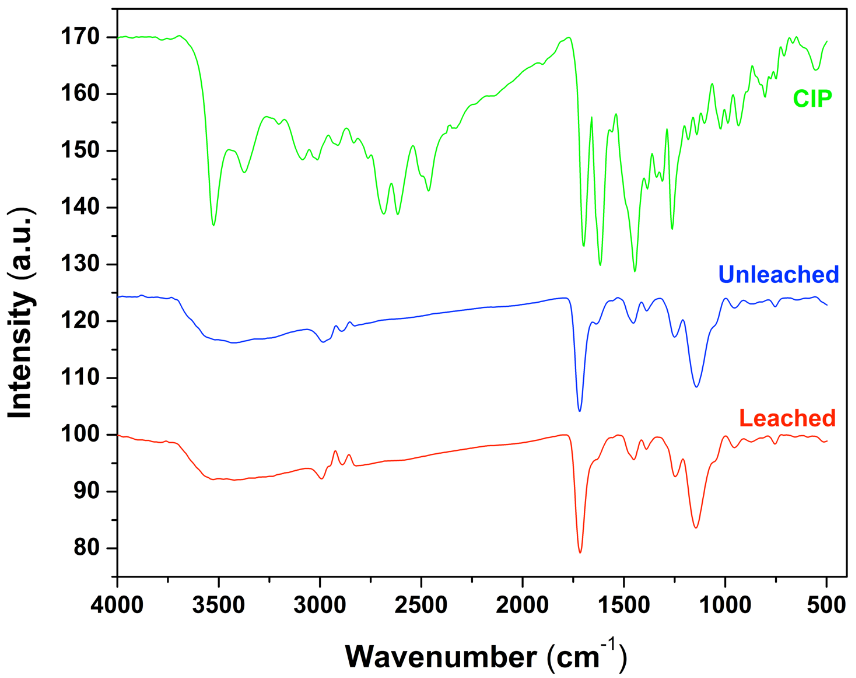

Figure S 1. FT-IR of ciprofloxacin (CIP), unleached and leached imprinted polymer

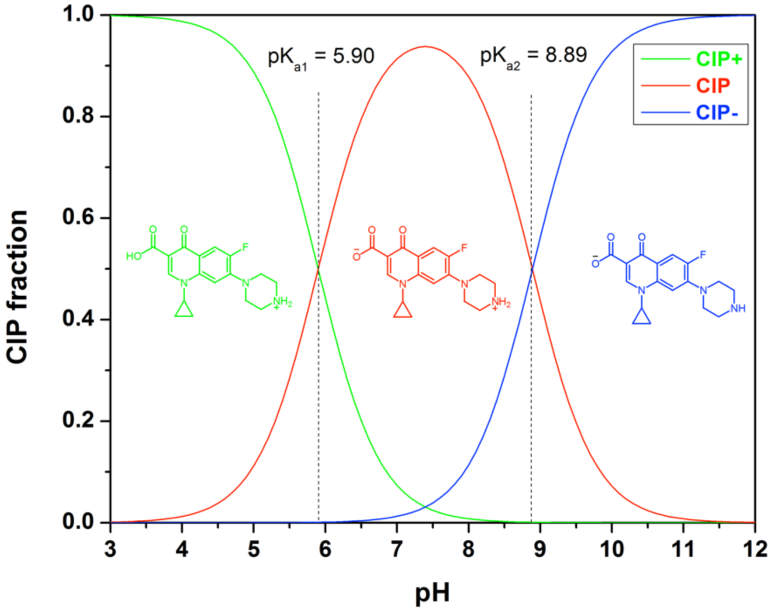

Figure S 2. Molecular structure of CIP and its ionic forms as a function of pH, calculated with pKa_1 = 5.90 and pKa_2 = 8.89

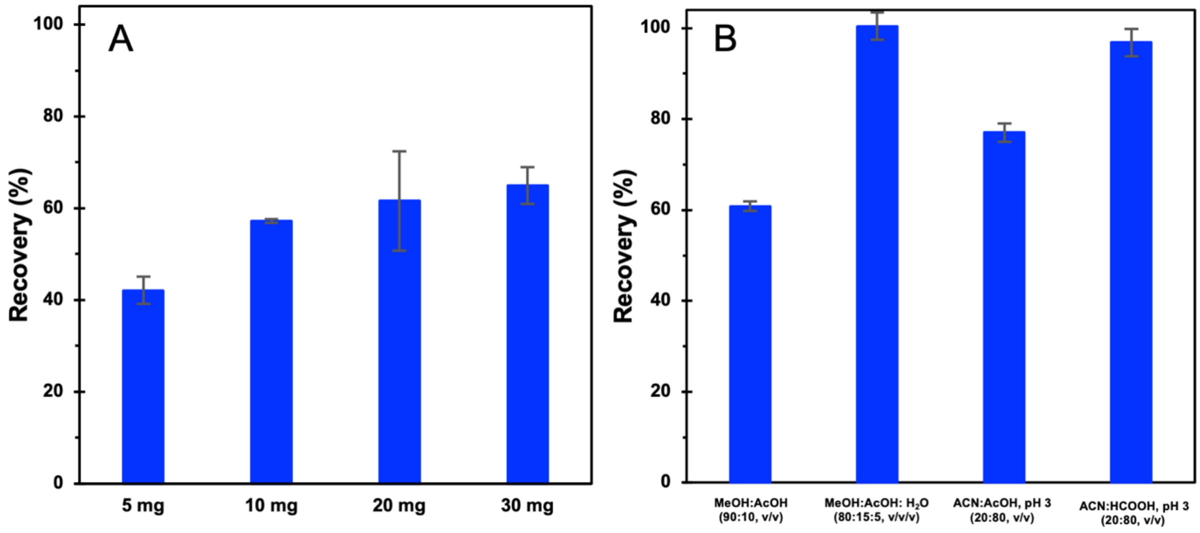

Figure S 3. Influence of mass of polymer (**A**) and elution solvent (**B**) on CIP extraction recovery

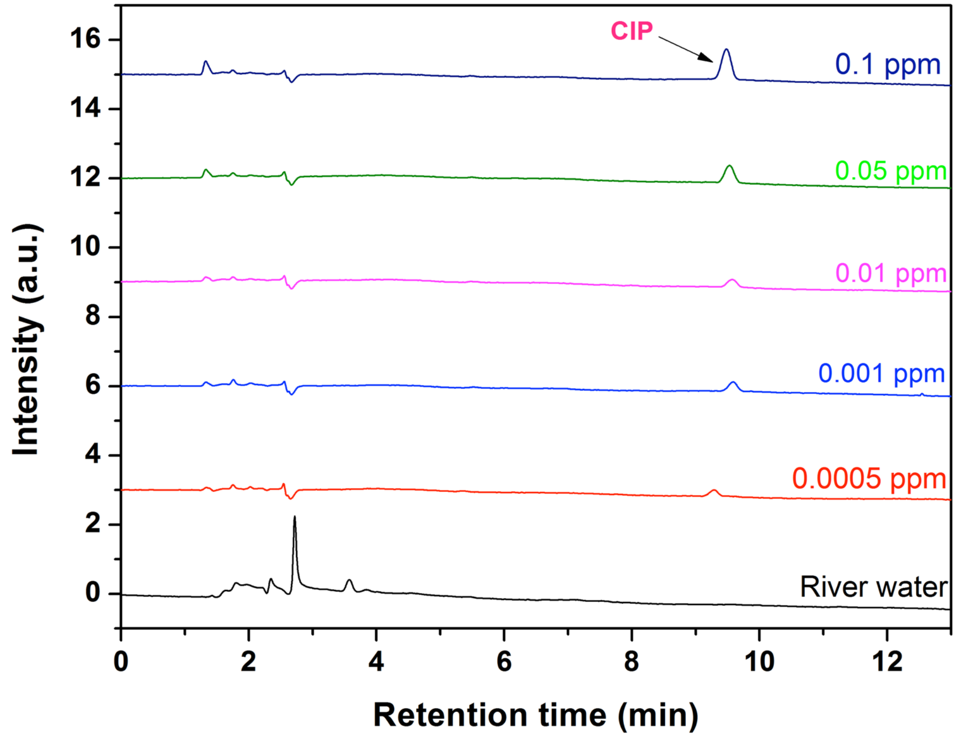

Figure S 4. Chromatograms of real river warter samples and extraction of spiked samples at diffrente concentrations

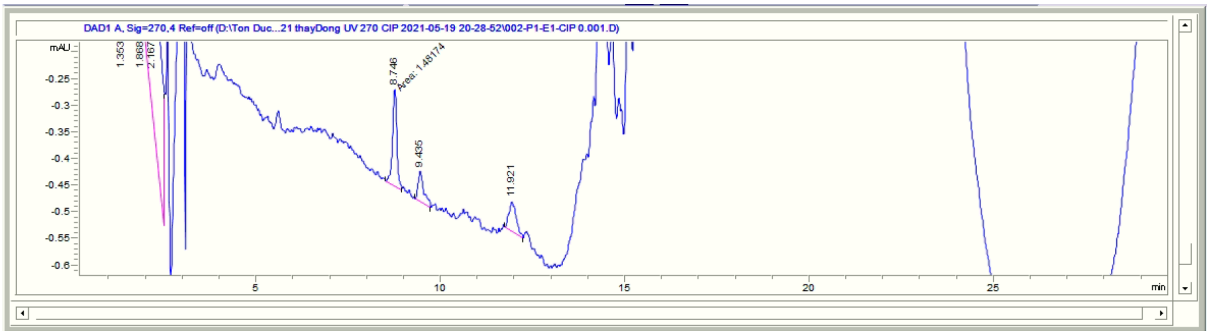

Noise determination:

Time range from [min]	to [min]	Noise (6*SD) [mAU]	Noise (PtoP) [mAU]	Noise (ASTM) [mAU]	Wander [mAU]	Drift [mAU/h]
6.000	7.000	2.395e-2	1.823e-2	-	-	-5.853e-1
10.000	11.000	3.011e-2	2.143e-2	-	-	-8.803e-1

RetTime [min]	k'	Area [mAU*s]	Height [mAU]	Symm.	Width [min]	Plates	Resolution	Signal /Noise
1.353	-	5.03349e-1	1.26174e-1	1.06	0.0650	2393	-	5.3
1.868	-	2.66074	3.53756e-1	1.23	0.1316	1120	3.08	14.8
2.167	-	14.13431	7.43272e-1	0.76	0.3396	225	0.74	31.0
8.746	-	1.48174	1.81036e-1	1.07	0.1167	31121	16.95	6.0

Figure S 5. Reported chromatogram of 0.001 ppm CIP standrad solution

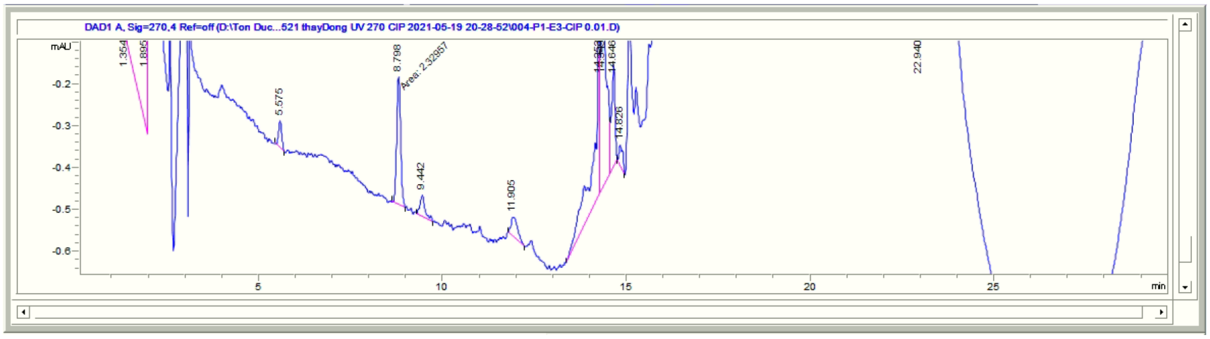

Noise determination:

Time range from [min]	to [min]	Noise (6*SD) [mAU]	Noise (PtoP) [mAU]	Noise (ASTM) [mAU]	Wander [mAU]	Drift [mAU/h]
6.000	7.000	2.205e-2	1.554e-2	-	-	-1.121
10.000	11.000	3.208e-2	2.229e-2	-	-	-1.056

RetTime [min]	k'	Area [mAU*s]	Height [mAU]	Symm.	Width [min]	Plates	Resolution	Signal /Noise
1.354	-	2.22928	2.77190e-1	1.41	0.1033	950	-	12.6
1.895	-	10.65650	6.67580e-1	3.41	0.5418	68	0.99	30.3
5.575	-	3.97873e-1	6.62914e-2	0.91	0.0944	19290	6.79	3.0
8.798	-	2.32957	3.07315e-1	0.92	0.1160	31880	18.00	9.6

Figure S 6. Reported chromatogram of 0.01 ppm CIP standrad solution

Table S 1. Pseudo-first-order and pseudo-second-order parameters for CIP adsorption on polymers MIP4 and NIP4

Polymer	Lagergren first-order			Pseudo second-order			Interparticle mass transfer diffusion	
	q_e (mg g^{-1})	k_1 (min^{-1})	R^2	q_e (mg g^{-1})	k_2 (mg g^{-1} min^{-1})	R^2	k_{id} (mg g^{-1} min^{-1})	R^2
MIP4	5.1103	0.0988	0.9740	5.4371	0.0361	0.9926	0.1425	0.9710
NIP4	4.3379	0.0629	0.9312	4.8370	0.0183	0.9391	0.2610	0.9344

Table S 2 : Interaction configurations of methacrylic acid (MAA) with CIP molecule

Interaction energies E_{int} (kJ/mol)	-72.12	-36.24	-19.30
Configurations	I- Double hydrogen bonds between carboxylic groups 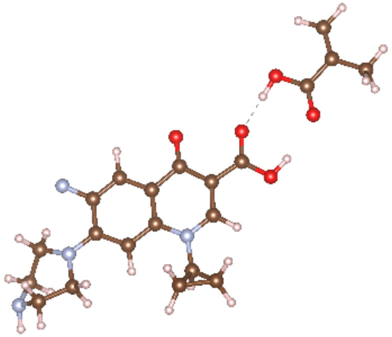	II- Hydrogen bond between H atom of MAA carboxylic group and carbonyl group of CIP 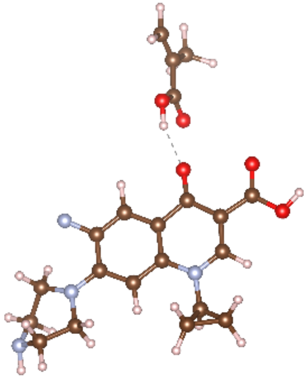	III- Hydrogen bond between H atom of MAA carboxylic group and fluorine atom of CIP 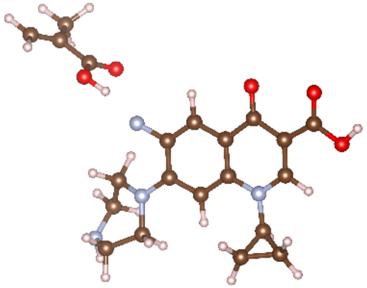

-55.55	-25.98
IV- Hydrogen bond between H of MAA carboxylic group and one nitrogen atom of CIP 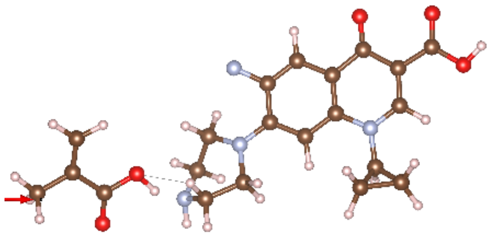	V- flat interaction between MAA and CIP 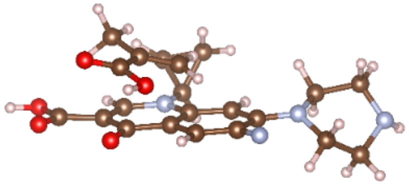

Table S 3 : Interaction configurations of 2-vinylpyridine (2-VP) with CIP molecule

Interaction energies E_{int} (kJ/mol)	-48.96	-53.43	-19.58
Configurations	I- Hydrogen bond between H atom of carboxylic group and nitrogen atom of 2-VP 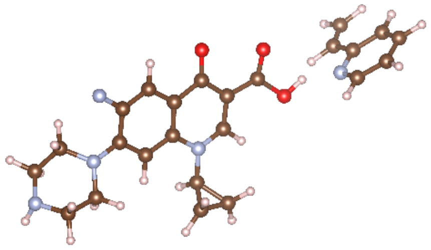	II- Pi-pi stacking between benzyl groups of CIP and 2-VP 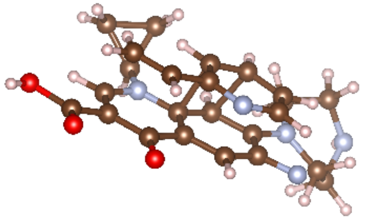	III- Hydrogen bond between H atom of ethylene group of 2-VP and oxygen atoms of CIP 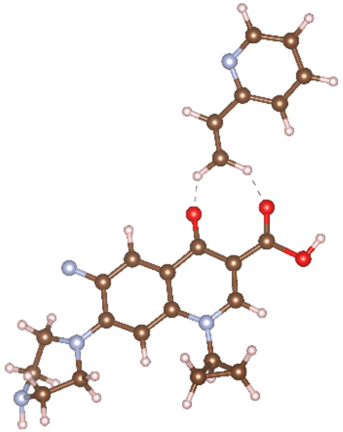

-8.46	-33.30
IV- Hydrogen bond between H atom of ethylene group of 2-VP and nitrogen atom of CIP 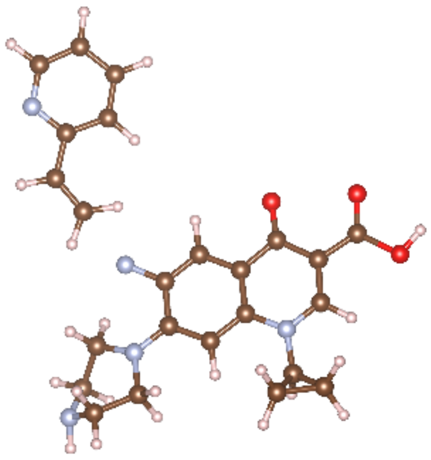	V- Hydrogen bond between N atom of 2-VP and h atom bonded to nitrogen atom in CIP 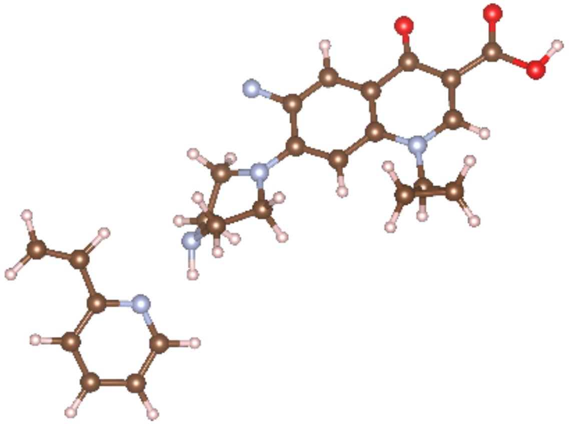

Table S 4 : Monomer-monomer interactions

Interaction energies E_{int} (kJ/mol)	**-50.88**	-6.00	-17.30
Configurations	I- H bond between 2VP nitrogen atom and H of MAA carboxylic group 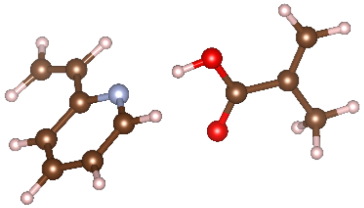	II- H bond between H atom of ethylene of 2VP atom and O atom of MAA carboxylic group 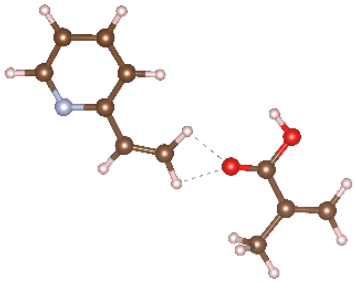	III- Flat interaction between MAA and 2VP 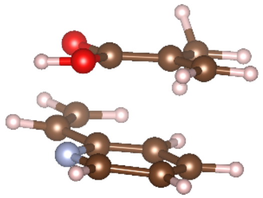

Table S 5 : Monomers arrangement in MIP and NIP polymers

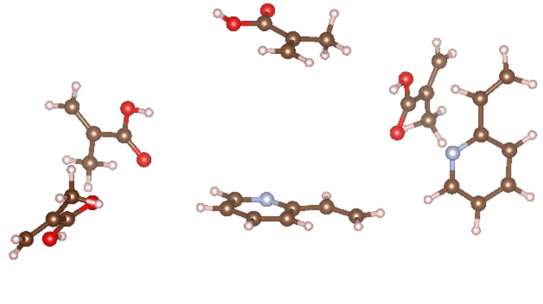	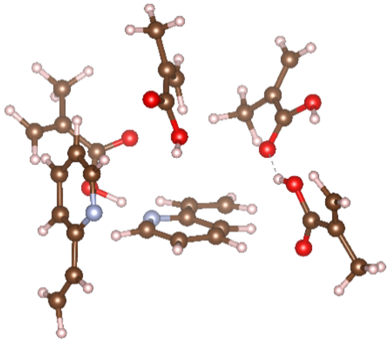
I. MIP	II. NIP
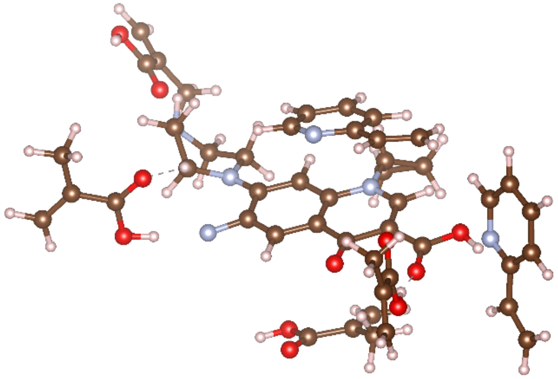	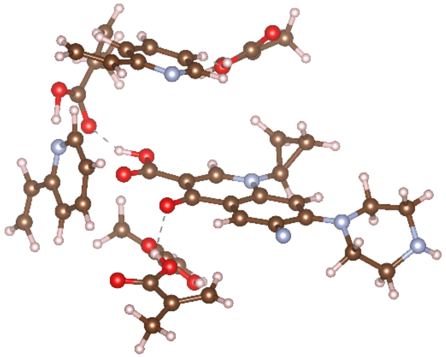
III. CIP-MIP	IV. CIP-NIP
